# Rethinking nutritional villains: A trans‐fatty acid identified to boost immunotherapy

**DOI:** 10.1002/ctm2.1537

**Published:** 2024-01-22

**Authors:** Hao Fan, Freya Q. Zhang, Jing Chen

**Affiliations:** ^1^ Department of Medicine The University of Chicago Chicago Illinois USA

**Keywords:** CD8+ T cell, immunotherapy, trans‐vaccenic acid

Among the myriad dietary nutrients, trans‐fatty acids or trans‐fats have long been vilified due to their association with cardiovascular diseases and other health risks.^1^ However, this paradigm has been challenged by emerging research suggesting a more nuanced role for certain trans‐fats.^2^ Our latest findings unveiled that trans‐vaccenic acid (TVA), a long‐chain trans‐fatty acid found in meat such as beef and lamb, and dairy products, reprograms CD8^+^ T cell function and enhances T‐cell based anti‐tumor immune response.^3^ With this commentary, we would like to delve deeper into the implications of this discovery, underscoring the potential of TVA in supplementing current clinical approaches to cancer and redefining our understanding of dietary fats in health and disease.

Trans‐fats encompass a diverse group of unsaturated fats with varying sources and compositions. Industrially produced trans‐fats, which are from hydrogenated vegetable oils, commonly used in processed foods for their texture and shelf‐life, have often been associated with adverse health outcomes like heart disease and increased insulin resistance.^2^ While emerging research suggests that natural trans‐fats found in dairy and meat from ruminant animals may not exhibit the same effects as their artificial counterparts.^2^ In fact, some clinical studies indicate potential health benefits of these animal trans fats, such as improved metabolism and reduced risks for cardiovascular disease.^4^, ^5^ This evolving understanding reflects the nuanced nature of trans‐fats in health and disease that has not been comprehensively studied. The beneficial effects of trans fats may have been overlooked highlighting the need for deeper investigation into the diverse roles of diet‐derived nutrients of distinct origins and structures.

How specific nutrients, including trans‐fats could influence specific biological processes is an underexplored area due to the complexity of cellular interactions and the diverse variables involved. To overcome this challenge, we compiled a blood nutrient library that includes 255 commercially available circulating dietary nutrients.^3^ This focused library enabled us to focus on the bioactivity of diverse nutrients despite their food origins and allowed us to perform high‐throughput cell‐based screens.

TVA emerged from our unbiased library screen as the top candidate that enhances the production of effector cytokines from T cells. We found that mechanistically, TVA antagonizes cell‐surface protein‐coupled receptor GPR43 and activates the cAMP‐PKA‐CREB axis, leading to enhanced effector CD8^+^ T cell function and anti‐tumor immunity. Moreover, syngeneic mice feeding on a TVA‐enriched diet show significant attenuation of the growth potential of immunogenic tumours. Our initial findings highlight the potential of TVA supplementation to enhance various T cell‐based cancer treatments (Figure 1).

**FIGURE 1:**
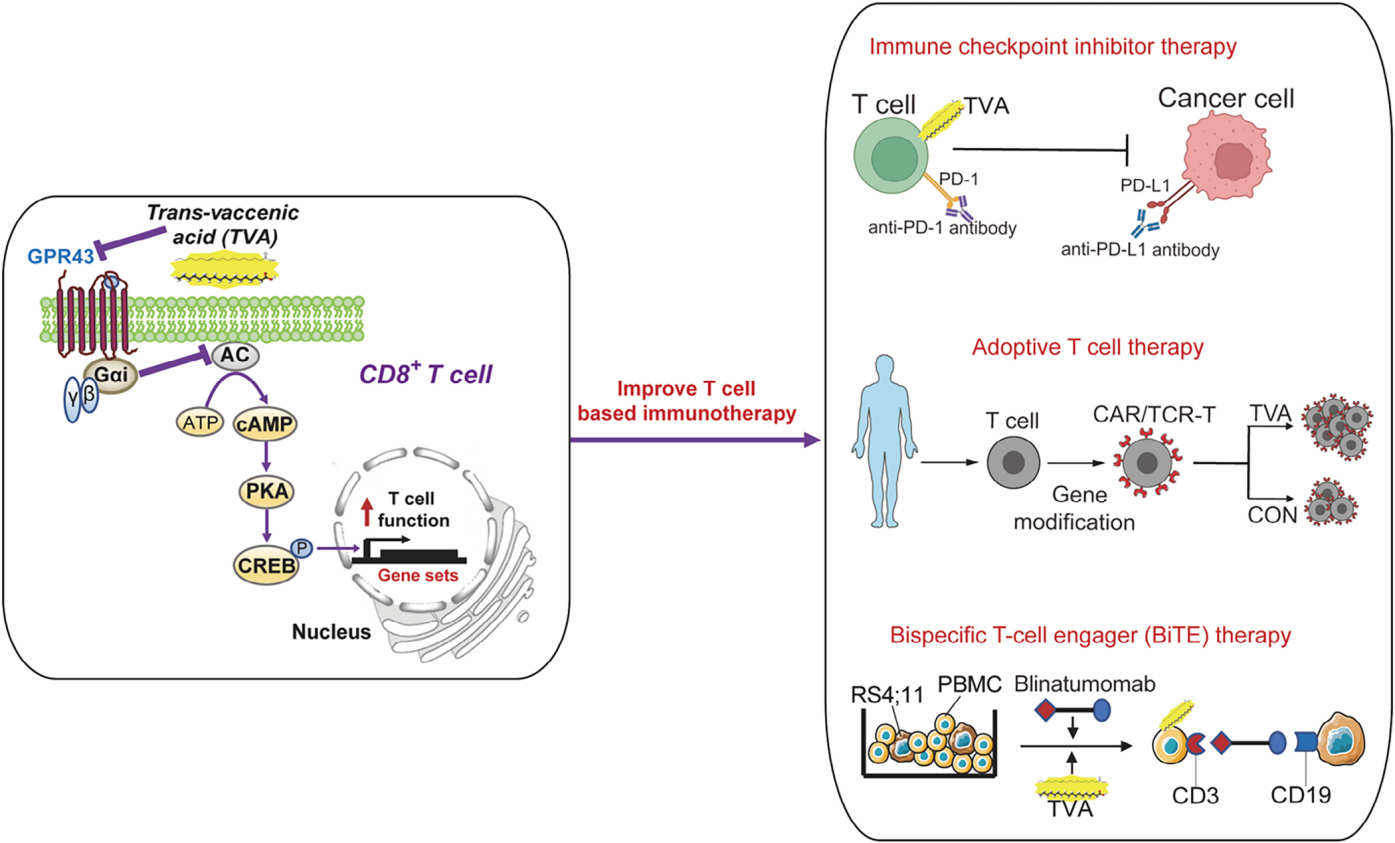
Working model of trans‐vaccenic acid (TVA) on CD8^+^ T cell and applications on T cell‐based immunotherapies.

## 1 IMMUNE CHECKPOINT INHIBITOR THERAPY

TVA treatment in vitro significantly reduces primary human CD8^+^ T cell exhaustion induced by programmed death ligand‐1 (PD‐L1) exposure. When combined with anti‐PD‐1 antibody therapy in vivo, dietary TVA supplementation results in a synergistic effect with immune checkpoint inhibitor (ICI) therapy in attenuating tumour growth. Together, these results suggest that TVA treatment could supplement ICI therapy by rescuing cytotoxic T cells from exhaustion for enhanced effector functions against cancer.

## 2 BISPECIFIC T CELL ENGAGER THERAPY

Bispecific T cell engagers (BiTEs) are engineered proteins that recruit T cells to cancer cells, facilitating more efficient T cell attack.^6^ We investigated the impact of TVA supplementation on blinatumomab, the first BiTE approved for treating acute lymphocytic leukaemia (ALL).^6^ Blinatumomab is a constructed monoclonal antibody targeting CD19 antigen on B cells and CD3 for T cells. Blinatumomab is extremely effective for minimal residual disease, but the response rate in relapsed disease is < 50% due to lack of T‐cell activation. (Figure 1). TVA treatment significantly enhanced the killing efficiency of blinatumomab towards human peripheral blood mononuclear cells (PBMCs) on human B‐ALL RS4;11 cells in a dose‐dependent manner.

## 3 ADOPTIVE T‐CELL THERAPY

Adoptive T‐cell therapy (ACT) involves the infusion of T cells that have been modified and expanded in the lab such as TCR gene‐engineered T cells. Among six therapies approved by the Food and Drug Administration since 2017, chimeric antigen receptor (CAR) T‐cell therapy is an established treatment for haematological malignancies.^7^ After TVA treatment, CAR‐T cells derived from lymphoma patients aged between 42 and 47 years old showed increased in vitro expansion. However, CAR‐T cells derived from an elder patient (72 years old) did not respond to TVA treatment. These findings highlight the need for further research into the potential age‐dependent effects of TVA in clinical settings. Furthermore, in a retrospective study, we found that TVA can be detected at higher levels in the serum collected from lymphoma patients who responded to CAR‐T cell therapy than from those who did not respond.

## 4 FUTURE PERSPECTIVES AND RESEARCH DIRECTIONS

The study suggests that TVA could be used as a dietary supplement to help various T cell‐based cancer treatments, and it is important to determine the optimized amount of the nutrient itself, not the food source. Our first discovery of TVA's immune‐regulatory function marks the beginning of exploring TVA's potential in clinical applications. We hope to provide more insights into more T cell‐based therapeutics.

Taken together, our recent findings uncovered a novel beneficial role of trans‐fatty acid and provided a mechanistic explanation of its function in selectively reprogramming cellular activity and its implications on immunotherapeutics against cancer. Moreover, our work contributes to a broader discourse on dietary nutrients as nutraceuticals, challenging the oversimplified classification of dietary nutrients as ‘good’ or ‘bad’, underscoring the physiological importance of a more nuanced understanding of their complex biological roles and potential therapeutic benefits in diverse clinical contexts.

## AUTHOR CONTRIBUTIONS

Hao Fan and Freya Q. Zhang have contributed equally to this work.

## CONFLICT OF INTEREST STATEMENT

The authors declare no conflict of interest.

## ETHICAL APPROVAL

Not applicable
